# Supplementary material for: Fine Carbohydrate Structure of Dietary Resistant Glucans Governs the Structure and Function of Human Gut Microbiota
Source: Nutrients. 2021 Aug 24;13(9):2924. doi: 10.3390/nu13092924 (PMC8467459; doi:10.3390/nu13092924)
Supplement: Supplementary file 1 [file nutrients-13-02924-s001.zip › nutrients-1320430-supplementary.pdf]

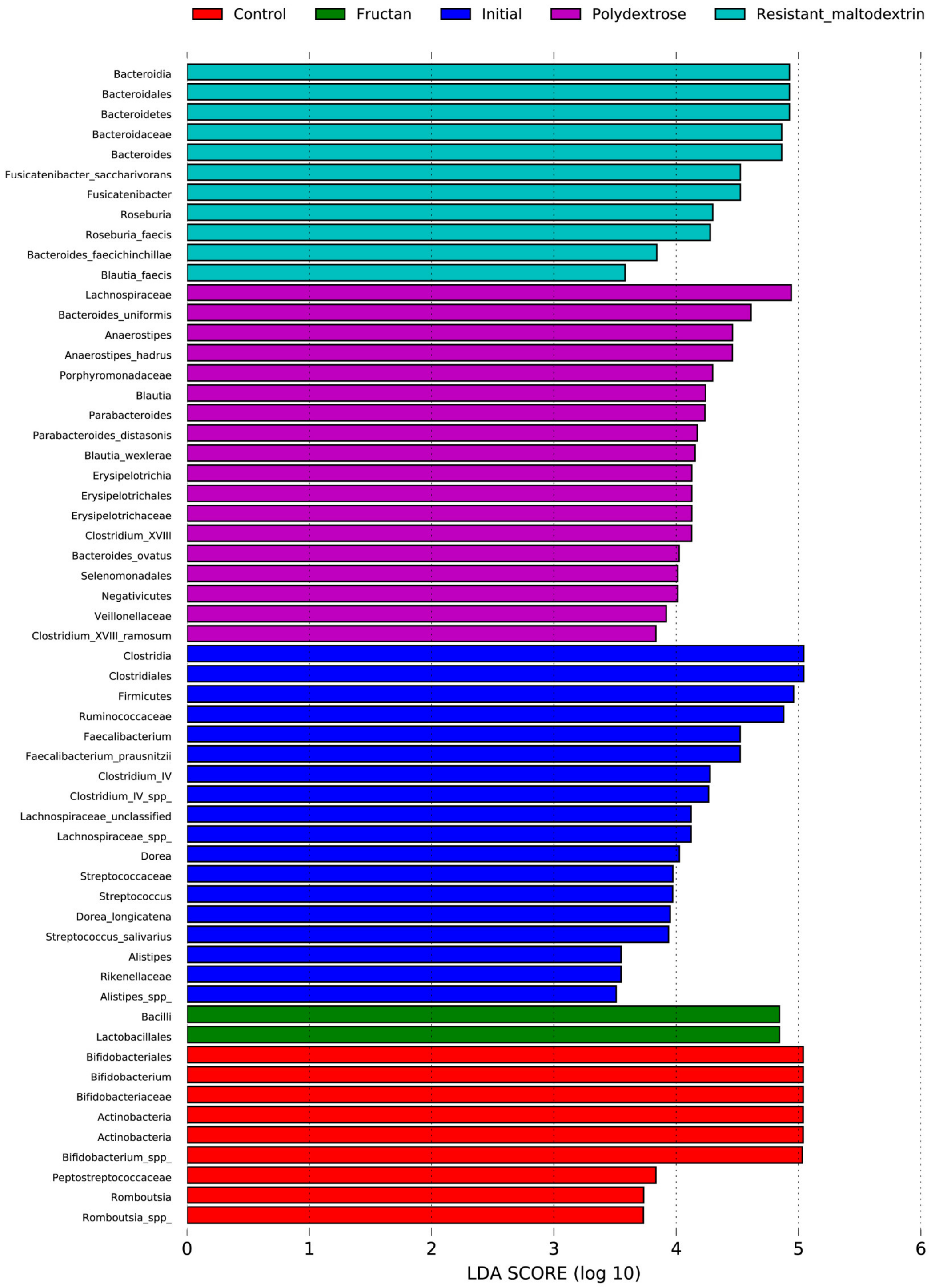

Figure S1. Linear discriminant analysis conducted with respect to glucan classes. Taxa with LDA scores ≥ 3.5 are shown.

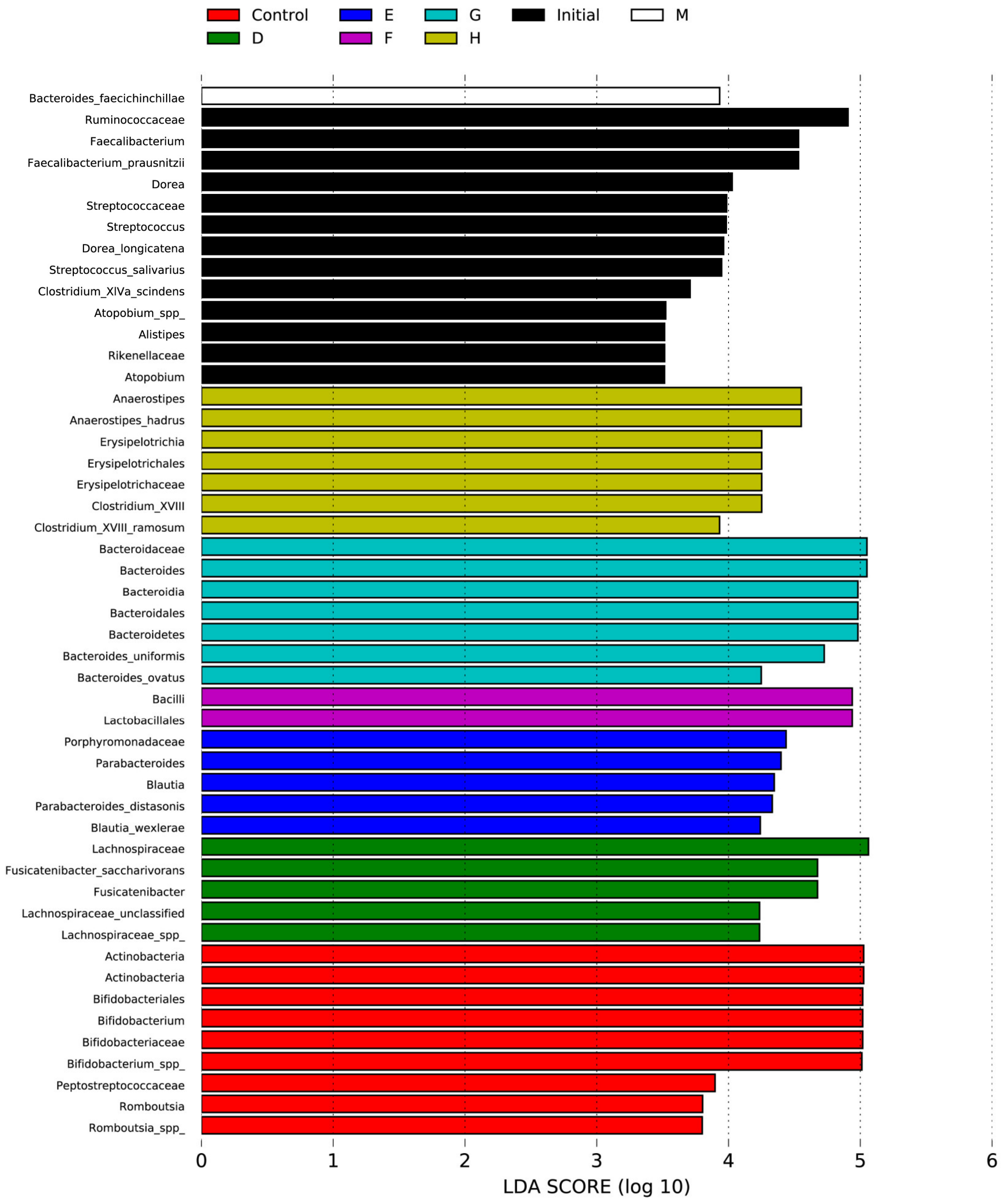

Figure S2. Linear discriminant analysis conducted with respect to individual glucans. Taxa with LDA scores ≥ 3.5 are shown.

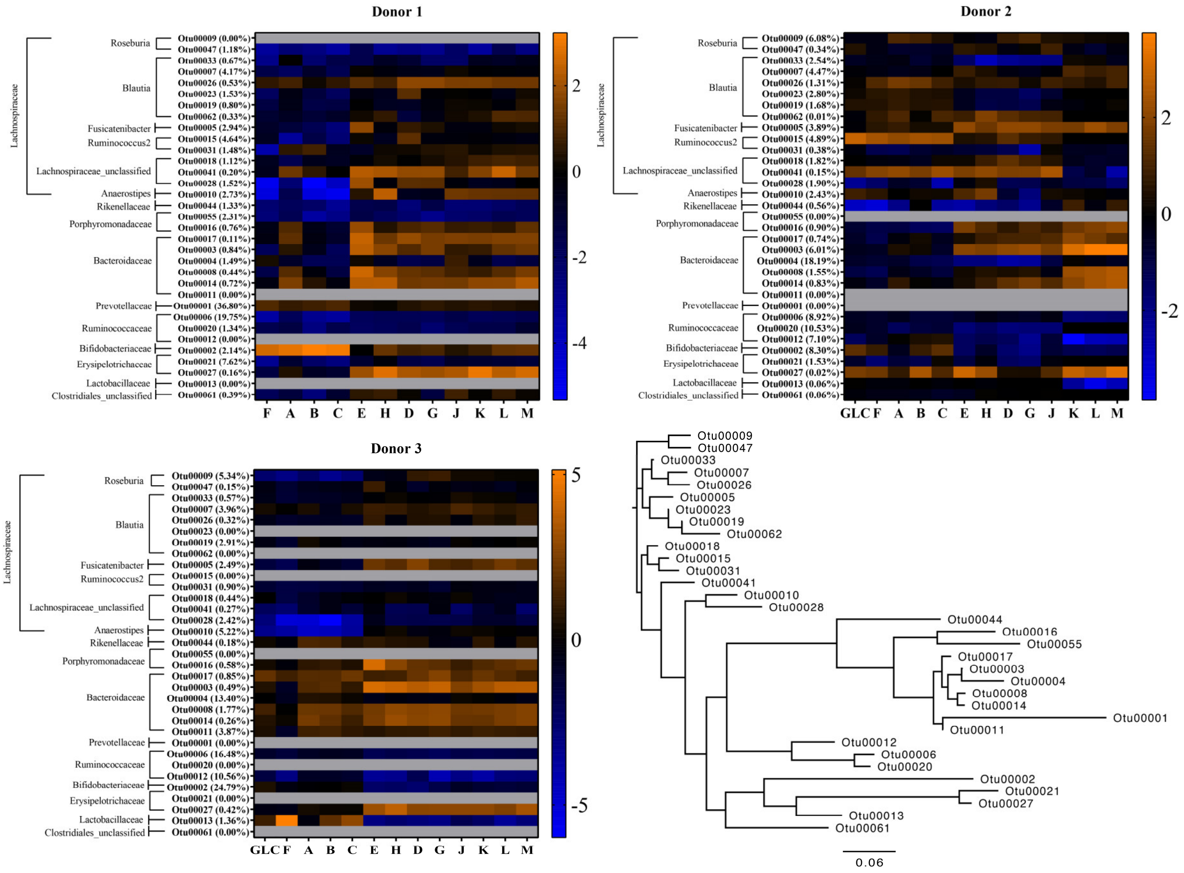

Figure S3. Log2-transformed changes in relative abundance over 48 hours of fermentation (abundance in 48 hr culture divided by that in initial fecal inoculum) for the most-abundant 33 OTUs across donors. Initial abundances for each donor are noted in parentheses next to the OTU number. Bottom right: phylogenetic relationships of OTUs generated using Fasttree.
